# Unraveling the message: insights into comparative genomics of the naked mole-rat

**DOI:** 10.1007/s00335-016-9648-5

**Published:** 2016-06-30

**Authors:** Kaitlyn N. Lewis, Ilya Soifer, Eugene Melamud, Margaret Roy, R. Scott McIsaac, Matthew Hibbs, Rochelle Buffenstein

**Affiliations:** Calico Life Sciences LLC, 1170 Veterans Blvd, South San Francisco, CA 94080 USA; Computer Science Department, Trinity University, San Antonio, TX 78212 USA

## Abstract

Animals have evolved to survive, and even thrive, in different environments. Genetic adaptations may have indirectly created phenotypes that also resulted in a longer lifespan. One example of this phenomenon is the preternaturally long-lived naked mole-rat. This strictly subterranean rodent tolerates hypoxia, hypercapnia, and soil-based toxins. Naked mole-rats also exhibit pronounced resistance to cancer and an attenuated decline of many physiological characteristics that often decline as mammals age. Elucidating mechanisms that give rise to their unique phenotypes will lead to better understanding of subterranean ecophysiology and biology of aging. Comparative genomics could be a useful tool in this regard. Since the publication of a naked mole-rat genome assembly in 2011, analyses of genomic and transcriptomic data have enabled a clearer understanding of mole-rat evolutionary history and suggested molecular pathways (e.g., NRF2-signaling activation and DNA damage repair mechanisms) that may explain the extraordinarily longevity and unique health traits of this species. However, careful scrutiny and re-analysis suggest that some identified features result from incorrect or imprecise annotation and assembly of the naked mole-rat genome: in addition, some of these conclusions (e.g., genes involved in cancer resistance and hairlessness) are rejected when the analysis includes additional, more closely related species. We describe how the combination of better study design, improved genomic sequencing techniques, and new bioinformatic and data analytical tools will improve comparative genomics and ultimately bridge the gap between traditional model and nonmodel organisms.

“From elephant to butyric acid bacterium—it is all the same” (Kluyver and Donker 1926). Or, stated another way, “anything found to be true of *E. coli* must also be true of elephants” (Monod 1997). The use of animal models in biomedical research stems from the concept expounded from these famous adages. Indeed, there is a considerable unity in the genomic, molecular, and biochemical mechanisms across bacteria, fungi, worms, flies, mice, elephants, and humans. Most basic biological principles and genetic regulatory mechanisms are thought to have arisen very early in the evolutionary history, as they are similar in prokaryotes, as well as single-cell and multi-cellular eukaryotes. While there is conservation of key molecular pathways among all organisms, during evolution existing components may be rearranged to acquire novel and/or improved functions. For example, at the protein level, subtle differences in fetal versus adult hemoglobin sequences modulate oxygen affinity. Additionally, at the organ level, the development of a vulva in female roundworms provides a mechanism for oviposition and heterosexual reproduction, and this arose from tissue remodeling processes dependent upon numerous cell–cell interactions and intercellular signaling pathways [see (Sternberg 2005)]. Similarly, multiple solutions may have evolved to address specific needs (e.g., formation of different kinds of eyes from existing structures to perceive images and facilitate vision). It is clear that there are many ways involving molecular, structural, and/or enzymatic components of achieving a particular function or organ. Such evolutionary tinkering and the concomitant altering or recycling of various components have contributed to integrated adaptations through the process of natural selection (Jacob 1977). It is likely that the tremendous diversity in species lifespan has also arisen as a consequence of evolutionary fiddling when modulation of certain processes may indirectly affect lifespan (Jacob, 1977).

While gymnosperms currently hold the longevity record for living organisms [e.g., the tree tumbo *Welwitschia mirabilis* (1500 years; Misra et al. 2015) and the bristlecone pine *Pinus longaeva* (5000 years; Brutovská et al. 2013)]; animal lifespans span an approximately 60,000-fold range. Mayflies and gastrotrichs live a mere 3 days and the ocean quahog (hard clam) lives >500 years (Carey 2002; Butler et al. 2013). Differences in species maximum lifespan are most apparent when comparing those species living in extreme environments with those living in more favorable, protected, and stable environments; animals living in ephemeral, temporary ponds (e.g., killifish) complete their lifecycle in a matter of days/weeks (Valdesalici and Cellerino 2003), while those living in more stable habitats such as the ocean [e.g., quahog (Butler et al. 2013) and bowhead whale (Craig George and Bockstoce 2008)] can live for centuries, possibly because they are subject to more relaxed evolutionary pressures. Animals living in different niches, particularly those habitats considered harsh, are likely to have evolved distinct mechanisms favoring their survival in that milieu. If not, that particular species would become extinct. Many of these ecophysiological adaptations may also influence species lifespan (Sanchez et al. 2015).

Large differences in species maximum lifespan potential [MLSP] must ultimately be genetically encoded; however, if a specific “lifespan program” existed, one might expect that genetic revertants of such a program could be identified to enable immortality. To date, no such observation has been made. So while it is highly unlikely that age of death is programmed, genetic regulation of the many pathways that contribute to survival of the individual (e.g., resistance to stress, damage eradication, and/or somatic repair), as well as genetic regulation of the metabolic pathways that inflict age-related damage, is likely to be directly involved in organismal longevity (Gems and Partridge 2013).

Observations based on “natural evolutionary experimentation” may elucidate mechanisms explaining how some species are able to live healthier and longer lives than others. Comparative biology may also reveal whether or not a mechanism is unique to a species (i.e., a private mechanism) or ubiquitously shared (i.e., a public mechanism) across evolutionarily distinct clades (Martin 1997; Partridge and Gems 2002). Comparative genomics is a relatively new field in which the comparison of genome sequences is used to identify candidate genetic variants associated with particular traits of interest (Alfoldi and Lindblad-Toh 2013). While elements of comparative genomics can potentially help identify genetic factors that contribute to extreme longevity, the lack of high-quality genomes, and the large evolutionary distances amongst species pose difficult challenges to overcome in the search for genetic determinants that modulate aging.

## Evolution of genes that modulate longevity

Comparative genomics is a powerful tool that exploits millions of years of evolution to identify the natural mechanisms that may have led not only to prolonged longevity, but also to different phenotypes associated with disparate resistance to cancer and other diseases. These genomic variations, rooted in evolution, are likely to be well conserved and possibly directly pertinent to human health and lifespan. In keeping with the maxim of the Nobel laureate August Krogh, that for every biological question there is an animal model ideally suited to tackle that research focus (Jorgensen 2001), naturally long-lived species—like the naked mole-rat—are prime candidates for identifying mechanisms involved in both delaying the onset, and slowing the rate, of aging.

Aging and longevity research has relied extensively on a battery of commonly used and relatively short-lived eukaryote model organisms, namely yeast, worms, flies, and fish, as well as mice and rats, to explore both genetic and environmental determinants of lifespan. While these short-lived models have each yielded a number of fascinating findings and insights into hypotheses surrounding extended lifespan and healthspan, they may also have constrained this complex, multifactorial field to areas in which they are best suited, most notably short-term intervention studies and genetic manipulations. Studies based upon these organisms revealed that changes in even a single gene (e.g., *age*-*1*, phosphatidylinositol 3 kinase) can extend lifespan of *Caenorhabditis elegans* (Friedman and Johnson 1988). Similar lifespan extension effects are evident in flies and mice when the insulin/IGF, gastric hormone, and the *Nrf2/skn*-*1* detoxification/xenobiotic pathways are genetically manipulated (Kenyon et al. 1993; Brown-Borg et al. 1996; Morris et al. 1996; Clancy et al. 2001; An and Blackwell 2003; Sykiotis and Bohmann 2008; Selman and Withers 2011; Ziv and Hu 2011). Furthermore, various types of dietary restrictions, whether limiting access to calories or amino acids, generally have a conserved effect of enhancing longevity across model systems (McCay et al. 1935; Klass 1977; Weindruch and Walford 1982; Jiang 2000; Selman and Withers 2011; McIsaac et al. 2016), although exceptions do exist (Liao et al. 2010). Collectively, these data support the premise that longevity can be modulated, likely through the regulation of nutrient signaling and stress response, which in turn impacts development, growth, reproduction, and survival. Strikingly, monozygotic human twins, as well as genetically identical individuals of these animal models (e.g., C57BL/6 mice), even when housed in the same environment and fed the same diet do not all have the same lifespans, suggesting that stochastic factors and epigenetic drift influence the hazard rate (i.e., the risk of death as it changes over a lifespan) and subsequent mortality (Finch and Kirkwood 2000; Herndon et al. 2002; Fraga et al. 2005).

Collectively, these findings contribute to the many convincing arguments that death and/or aging are neither genetically programmed nor under evolutionary selection pressure (Martin 1997; Partridge and Gems 2002; Kirkwood and Melov 2011). Rather, they are due to co-evolution with other traits. For example, short- and long-lived organisms may exhibit different responses to changes in the environment and thereby indirectly affect survival. Indeed the various nutrient-sensing genes shown to modulate longevity (e.g., insulin/IGF-1, FOXO, mTOR) appear to regulate resource allocation for somatic maintenance and thereby influence survival [reviewed in (Kapahi 2010)].

## Mammalian models of aging

The mouse is the most widely used mammalian model in biomedical research. Raised in cages in protected vivaria, they are not subjected to typical natural selective pressures such as predation or food limitation. In the wild, we contend that rodents are unlikely to die from age-associated disease linked to genomic instability (e.g., cancer) or a disruption in proteostasis (e.g., proteinopathies), as they do in the laboratory. Rather, their death in the wild more commonly results directly from stochastic and random events (e.g., predation, extreme weather conditions leading to starvation, and/or infections) (Collins and Kays 2014). As such, laboratory mice are more like sedentary humans, and are likely to provide many insights into a first world western lifestyle.

The evolutionary theory of aging posits that longevity assurance mechanisms may have evolved in those species that have low extrinsic mortality, such as animals that (a) live underground and are protected from climatic extremes, germs, and predation (e.g., naked mole-rats), (b) can escape hostile habitats (e.g., bats and birds), or (c) have effective body armor (e.g., porcupines and tortoises) (Chen and Maklakov 2012). As such, mice, which experience high extrinsic mortality in the wild and have shorter lifespans than predicted on the basis of body size in captivity, may not necessarily be the best organism with which to search for longevity assurance mechanisms (Buffenstein et al. 2014).

Moreover, as organisms become less fecund with age, the forces governing natural selection decline and the genetic factors that influence somatic maintenance and organismal survival in the face of stochastic damage likely play a greater role in the determination of survival and longevity of the organism (Hamilton 1966). Greater selection pressures that enhance somatic maintenance—thereby extending the time taken before accrued damage can induce a significant decline in function and viability—may also extend the lifespan of the animal. It is thus highly likely that long-lived species employ different mechanisms to those of short-lived species to defend their soma and are therefore useful animal models to address the evolved mechanisms involved in retarding the aging process and extending longevity, particularly of other long-lived species, like humans. One such animal model of exceptional biogerontological interest is the naked mole-rat (Austad 2009).

## Unusual features of the long-lived naked mole-rat

**Fig. 1:**
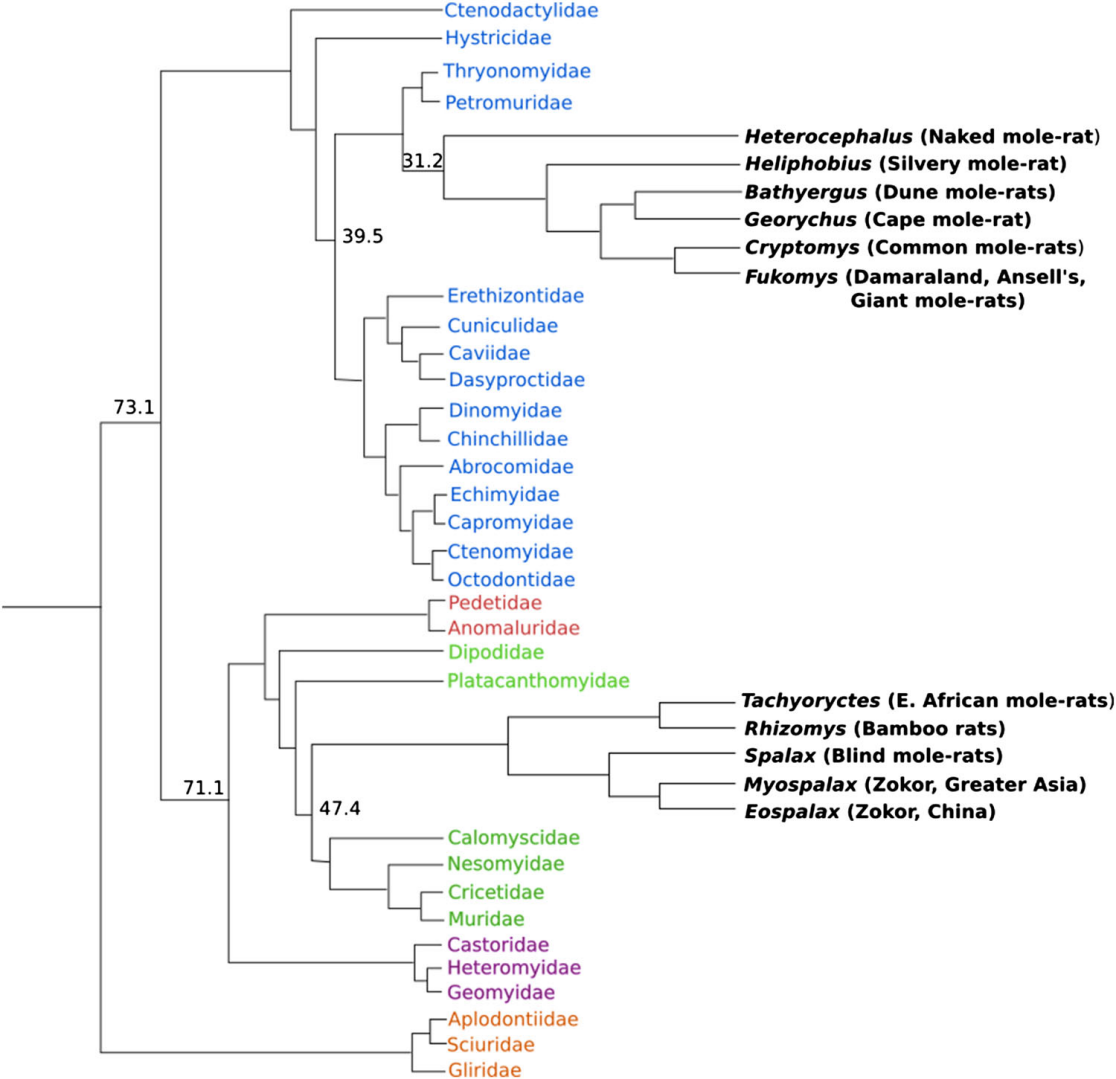
Phylogenetic relationships of rodent species: the Bathyergidae, Heterocephalidae, and Spalacidae families are in two different suborders of Rodentia. Genome data are often compared between naked mole-rats and mice; however, mice are more closely related to blind mole-rats (*Spalax*), which are in the Muroidea superfamily. Naked mole-rats are more closely related to guinea pigs (Ctenophiomorpha), both species diverged ~39.5 million years ago (mya). Naked mole-rats and mice diverged from their common ancestor ~73.1 mya (and blind mole-rats from mice ~47.4 mya). Additionally, mice appear to be evolving faster than either of the mole-rat species, which could account for many of the differences observed between these unique organisms

The naked mole-rat is only one of over 50 subterranean dwelling rodents found throughout the world (Begall et al. 2007; Table 1). This species belongs to the Ctenohystrica supraorder of rodents, made up of the superfamilies Phiomorpha (African mole-rats, rock rats, and porcupines) and Caviomorpha (tuco tucos, degus, and guinea pigs). Recently, it was concluded that naked mole-rats diverged 31 million years ago [mya] prior to the diversification of other African mole-rat species (the Bathyergidae family), and the naked mole-rat has been now placed in a separate family (Heterocephalidae; Fig. 1) (Faulkes et al. 2004; Patterson and Upham 2014). Among mammals, only the Heterocephalidae (the naked mole-rat) and Bathyergidae (African mole-rats, i.e., the long-lived Damaraland mole-rat [MLSP 20y; Buffenstein *pers. com*]) include species that are considered truly eusocial (Bennett and Faulkes 2000); in that, similar to the eusocial insects (e.g., wasps, bees, and ants) there is a well-defined social hierarchy in which breeding is restricted to a single female (“the queen”) within the colony.

**Table 1:** Characteristics of mole-rats (data summarized from Lacey 2000; Begall et al. 2007)

Species	Common name	MLSP (years)	Body mass (g)	Haplotype (2n)	Social/Solitary	Reproduction	Ecology
*Heterocephalus glaber*	Naked mole-rat	31	40	60	Eusocial (up to 295 individuals/colony)	Gestation is 66–74 days. Up to 4 litters/year. 1–29 pups/litter	Found in the arid and semi-arid regions of north east Africa, in sandy soils that become hard during the dry season, and in areas with low and unpredictable rainfall
*Fukomys damarensis*	Damaraland mole-rat	16–20.6	60–600	80	Eusocial/Social 4 to over 45 individuals/colony, with the eusocial *F. damarensis* having the largest colonies. All other species social	Gestation ranges between 78 and 111 days. Up to 4 litters/year *F. damarensis* = 78–92 days, 1–6 pups/litter	Found all over sub-Saharan African living in a wide range of soil types and annual rainfall patterns
*Fukomys mechowii*	Giant mole-rat			40			
*Fukomys anselli*	Ansell’s mole-rat			69			
*Fukomys amatus*	Zambian mole-rat			68			
*Fukomys bocagei*	Angolian mole-rat			58			
*Fukomys darlingi*	Mashona mole-rat			54			
*Fukomys ilariae*	Somali striped mole-rat			Unknown			
*Fukomys kafuensis*	Kafue mole-rat			42–58			
*Fukomys vandewoestijneae*	Caroline’s mole-rat			44			
*Fukomys micklemi*	Kataba mole-rat			42–68			
*Fukomys whytei*	Malawian mole-rat			46			
*Fukomys foxi*	Nigerian mole-rat			Unknown			
*Fukomys zechi*	Togo(Ghana) mole-rat			Unknown			
*Fukomys ochraceocinereus*	Ochre mole-rat			Unknown			
*Cryptomys h. hottentotus* ^a^	Common mole-rat^a^	11	57–110	54	Social, 8–16 individuals/colony	Gestation is 59–66 days. Up to 2 litters/year. 2–4 pups/litter	Found in South Africa in a wide range of soil types and annual rainfall patterns
*Heliophobius argenteocinereus*	Silvery mole-rat	>7.5	160	62	Solitary, aggressive	Gestation is 87 days. 2–4 pups/litter	Lives in sandy soils of savannahs and woodlands with high annual rainfall
*Georychus capensis*	Cape mole-rat	11.2	180	54	Solitary	Gestation is 44–48 days. Up to 2 litters/year. 4–10 pups/litter	Found in sandy and clay soils in mesic habitats with winter rainfall patterns
*Bathyergus janetta*	Namaqua dune mole-rat	>6	Up to 1000	54	Solitary	Gestation is 52 days. Up to 2 litters/year. 1–7 pups/litter	Found along southwestern African coastal sand dunes. *B. suillus* is found in areas of high rainfall, and *B. janetta* in areas with very low rainfall
*Bathyergus suillus*	Cape dune mole-rat			56			
*Spalax ehrenbergi* ^b^	Blind mole-rat^b^	20.2	120	52–60	Solitary, varying levels of aggression	Gestation is 34 days. 1 litter/year. 1–5 pups/litter	Dozens of different biological species live in different climates and with distinct haplotypes distributed throughout the middle east
*Myospalax baileyi*	Plateau zokor	Unknown	Up to 720	Unknown	Solitary	Unknown	Distributed throughout Asia
*Myospalax myospalax*	Siberian zokor						
*Myospalax psilurus*	Transbaikal zokor						
*Eospalax fontanierii*	Chinese zokor	Unknown	Up to 600	Unknown	Solitary	Unknown	Distributed throughout mountainous regions of China
*Eospalax rothschildi*	Rothschild’s zokor						
*Eospalax smithii*	Smith’s zokor						
*Tachyoryctes splendens* ^c^	East African mole-rat^c^	>3.1	220	48	Solitary	Gestation is 36–41 days. Up to 2 litters/year. 1–3 pups/litter	Located in east and central Africa in multiple habitats
*Tachyoryctes macrocephalus*	Big-headed mole-rat						
*Rhizomys sinensis*	Chinese bamboo rat	>4	Up to 4000	Unknown	Solitary	Unknown	Distributed throughout Asia with in a wide range of habitats
*Rhizomys sumatrensis*	Sumatra bamboo rat						
*Rhizomys pruinosus*	Hoary bamboo rat						
*Cannomys badius*	Lesser bamboo rat						

African mole-rats from the Bathyergid and Heterocephalid families are generally lumped together. They together with blind mole-rats and zokors from Spalacidae are all subterranean dwelling rodents. Social structure varies considerably within these families, with some species eusocial, social, or solitary. Naked, Damaraland, and blind mole-rats are the most heavily researched with regard to their genome sequencing, and other physiological and biochemical characteristics that may contribute to their long lifespans, and prolonged healthspans

^a^ *Cryptomys hottentotus* is made up of several subspecies. Only *C. hottentotus hottentotus* is described here, although subspecies share the same haplotype and are of a similar body size

^b^The blind mole-rats (referenced as the superspecies *Spalax ehrenbergi* here) also include a number of other *Spalax* species and subspecies

^c^ *Tachyoryctes splendens* includes several subspecies

All mole-rat species (collectively the Bathyergidae, Heterocephalidae, and Spalacidae families) occupy underground niches. Although some of these evolutionarily divergent species lead a solitary existence and others live communally, they share many morphological and physiological traits considered adaptive to life below ground (Tables 1, 2; Bennett and Faulkes 2000; Begall et al. 2007). All mole-rats are herbivores, with most species restricting feeding to plant components found solely below ground, namely bulbs and tubers (Bennett and Faulkes 2000). Moreover, all appear to have lower resting metabolic rates than their above-ground-dwelling counterparts and all appear to be extremely tolerant of hypoxia. All mole-rats studied to date are considered long-lived for their body size, living at least twice as long as predicted allometrically. This phenomenon is most pronounced in the naked mole-rat, which has a longevity quotient (ratio of the observed maximal lifespan to that predicted by body mass) of >4, similar to that observed in humans, another very long-living species (Buffenstein 2005).

**Table 2:** A summary of common phenotypic characteristics of subterranean rodents

Phenotype	Naked mole-rat	Damaraland mole-rat	Blind mole-rat	Mouse
Eye size (diameter mm)	~0.75	~1.5	Completely degenerate	~3
Retina	Regressive and disorganized	Irregularly structured	Vestigial and reorganiz ed	Strict organization
Detection of light images	Yes/no	Yes/no	Yes/no	Yes/can detect detailed image
External ear pinnae	No	No	No	Yes
Highly developed tactile vibrissae all over body	Yes	Yes	Yes	On face only
Internal testes in males	Yes	Yes	Yes	No
Mode of digging	Chisel tooth	Chisel tooth	Head–lift and chisel tooth	Feet
Strict Herbivores	Yes	Yes	Yes	No
Body temperature (°C)	33	35	35	37
Mass specific metabolic rate (% predicted by mass)	50	57	84	130
Thermal conductance (% predicted by mass)	254	134	150	100
Precision of thermoregulation	Predominantly thermally labile	Predominantly homeothermic	Predominantly homeothermic	Strictly homeothermic
NST capacity (fold increase relative to BMR)	4	2	3	2
Heart rate (beats per min)	200–370	150–300	110–200	350–850
Heart rate (% expected)	45	45	43	>120
Hematocrit (%)	46	48	45	38
Hemoglobin (g/dL)	14	14	15	12
Tolerance of hypoxia/hypercapnia	High	High	High	Low

Subterranean rodents share a number of features considered adaptations for life underground. The three mole-rat species (the naked, Damaraland, and blind mole-rat) highlighted here represent the Heterocephalidae, Bathyergidae, and Spalacidae families. All are morphologically streamlined (lack of ear pinnae and cryptorchidism), visually impaired with greater reliance on the somatosensory system. Linked to life in a sealed burrow system where gas exchange is restricted to diffusion through soil, all show reduced metabolic rates, heart rates, and oxygen consumption with concomitant changes in blood oxygen carrying capacity. Not surprisingly, mole-rats are resistant to hypoxia and hypercapnia; neither convective cooling nor evaporative water loss is particularly effective in humid sealed burrows, rather loss of metabolic heat is primarily facilitated by high rates of thermal conductance. Low metabolic rates coupled with high rates of thermal conductance give rise to lower resting body temperatures and less strict regulation of body temperature than observed in species that live above ground. Data obtained from (Bennett and Faulkes 2000; Lacey 2000; Cernuda-Cernuda et al. 2002; Begall et al. 2007)

The naked mole-rat is endemic to the arid and semi-arid regions of north east Africa (Sherman et al. 1991), living in large eusocial family groups in an extensive maze of sealed burrows ranging from 1 to 8 feet below the ground (Jarvis 1981). Having resided in this inhospitable underground milieu in sub-Saharan Africa since the early Miocene (~23 mya), naked mole-rats are extremely tolerant to a variety of conditions most other species cannot survive, including low partial pressures of oxygen and high amounts of carbon dioxide (Larson and Park 2009; Blass 2014). Naked mole-rats appear to be resistant to the highly poisonous allelochemicals (e.g., cyanide/glycosides) found in the plant storage organs they consume. They are also resistant to a wide variety of toxins, including heavy metals, DNA damaging agents, and chemotherapeutics (Salmon et al. 2008; Lewis et al. 2012), as well as to the toxic effects associated with the nitrogenous wastes (ammonia and methane) in their communal latrines (LaVinka and Park 2012). Collectively, these findings indicate a role for enhanced xenobiotic metabolism mechanisms in the long-lived naked mole-rat and other phylogenetically distant mole-rat species. The evolution of defenses against extrinsic mortality factors likely leads to protection against intrinsic factors linked to metabolic toxic moieties as well.

In vivaria, the naked mole-rat has a maximum captive lifespan that exceeds 30 years (Edrey et al. 2011a). For the better part of these three decades, naked mole-rats exhibit an extended healthspan and compression of the period of morbidity; they experience very little change in a number of physiological and biochemical characteristics that are typically associated with aging, including a sustained lean mass and well-maintained bone composition (O’Connor et al. 2002; Pinto et al. 2010), cardiac function (Grimes et al. 2012, 2014), basal metabolic rate (O’Connor et al. 2002), and proteome maintenance (Perez et al. 2009; Rodriguez et al. 2011). In addition to these distinctive features of slowed and attenuated aging, naked mole-rats are incredibly resistant to spontaneous neoplasia (Seluanov et al. 2009; Liang et al. 2010; Edrey et al. 2011a); over the last four decades of captive housing, reports of cancer are exceedingly rare. We have observed only one occurrence of naturally occurring cancer (a lymphoma in a 21-year-old female) in over 2000 necropsies. Two zoos recently reported rare instances of cancer in their long-maintained naked mole-rats (Delaney et al. 2016). Naked mole-rats show pronounced resistance to experimentally induced tumorigenesis (Liang et al. 2010) as well. In sharp contrast, cancer is often observed in C57BL/6 mice; even when cancer is not the direct cause of death, the majority of mice die with some signs of neoplastic lesions (Ikeno et al. 2009). Pronounced cancer vulnerability is thought to contribute significantly to the short lifespan of most laboratory mouse strains, which is approximately half of that predicted on the basis of body size (Hulbert et al. 2007).

Whereas in eusocial insects such as bees, the queens have maximum lifespans that are 2-10 fold longer than worker insects (Howell and Usinger 1933; Bozina 1961; Ribbands 1964; Wilson 1971; Seeley 1978; Hölldobler and Wilson 1990), the mole-rat breeding female (i.e., the colony’s queen) exhibits a lifespan similar to that of the subordinates in captive colonies (Buffenstein 2008). Although there are very few data on longevity of mole-rats in their native habitat, it has been reported that in the wild, with an army of subordinates to provide food and protection for the queens, the dominant breeders are found in the same burrow system for ~17 years, 4-fold longer than wild-living subordinates that may be preyed upon when foraging or may leave their natal colony during dispersal events (Begall et al. 2007).

Strikingly, the queen shows no decline in fertility with age and continues to produce offspring throughout her long life (Edrey et al. 2011b), with the ability to produce >1000 offspring during her “reign” (Buffenstein 2005). With only one breeding female and 1-4 breeding males in sealed underground habitats, these colonies not only remain relatively isolated from other populations of naked mole-rats, but also exhibit considerable inbreeding (Sherman et al. 1991). Indeed, DNA fingerprinting reveals that naked mole-rats have the highest coefficient of inbreeding (0.45) for any natural populations of mammals on record (Reeve et al. 1990). A small fraction (<1 %) of the mole-rat population belongs to the “dispersomorph” caste (animals that occasionally abandon their colony in search of a new colony) and are thought to play a critical role in increasing genetic heterogeneity (O’Riain et al. 1996; Clarke and Faulkes 1997; Faulkes et al. 1997).

## Genomics and transcriptomics of the naked mole-rat

Three studies have generated a significant amount of genomic (Kim et al. 2011; Keane et al. 2014,) and transcriptomic (Yu et al. 2011) data for the naked mole-rat. A handful of additional studies used these data to delve into the more unusual features of naked mole-rats (Kim et al. 2011; Fang et al. 2014; Davies et al. 2015). Here, we will discuss how methods of analysis, study design, and data quality can have a profound impact on effectively revealing mechanisms that might explain unique features of the naked mole-rat. We review these data and report on key findings as well as highlight areas where additional research and genomic analyses are needed.

### Genome assembly of the naked mole-rat

Two groups have independently published (Kim et al. 2011; Keane et al. 2015) draft assemblies of the naked mole-rat genome (Table 3). Both assemblies used shotgun whole-genome sequencing using high coverage Illumina data with a range of insert sizes. This approach is commonly used and has been applied to many species (Zerbino and Birney 2008; Li et al. 2010; Gnerre et al. 2011; Luo et al. 2012). The drawback of this approach, however, is that the assemblies are relatively fragmented and contain a high percentage (15 %) of unfilled gaps, hindering analyses of gene regulation and expression. Early short-read Illumina assemblies were shown to contain a significant number of misassemblies and may collapse homologous genes and/or pseudogenes (Zimin et al. 2012). With improvement of sequencing platforms and assemblies (e.g., long-read technologies for whole transcript sequencing (Sharon et al. 2013; Tilgner et al. 2014), as well as the ongoing assembly of many more genomes, the quality of data will continue to improve.

**Table 3:** Comparison of assembly statistics of commonly compared genomes of naked mole-rat, guinea pig, mouse, and human. Data from http://www.ncbi.nlm.nih.gov/genome/

	Naked mole-rat			Guinea pig	Mouse	Human
Genome	hetGla1	hetGla2	Cell Rep	cavPor3	GRCm38.p4	GRCh38.ph
Assembly size (Gb)	2.66	2.62	2.75	2.72	2.8	3.23
Sequencing coverage	92x	90x	92x	6.8x	N/A	N/A
# Contigs	273,990	114,653	N/A	61,604	796	1460
Contig N50 (Kb)	19.3	47.8	19.3	80.6	32,273	56,413
# Scaffolds	39,266	4229	N/A	3144	293	801
Scaffold N50 (Mb)	1585	20,533	21,307	27,942	52,589	59,364
Unfilled gaps (Mb)	214	303	N/A	60	79	161

Note that the naked mole-rat genome assemblies are less contiguous (lower contig N50) and more gapped (higher gap percentage)

Genomes of comparable species like mouse, rat, and guinea pig (Table 3), on the other hand, have been assembled by several research groups using multiple types of sequencing data (e.g., Illumina shotgun sequencing, Sanger sequencing, bacterial artificial chromosomes, etc.). Because of the inclusion of more types of data, these assemblies are significantly less fragmented and more complete than those of the naked mole-rat. In addition, these assemblies are constantly being updated using new technologies (Lander et al. 2001; Venter et al. 2001; Cheung et al. 2003; Consortium 2004; She et al. 2004; Chaisson et al. 2015). Comparing genomes with different levels of completeness presents a fundamental challenge, limiting both the data interpretations and the conclusions drawn. For example, since better resolution of repetitive elements is expected when using longer reads, the observation of a lower abundance of repetitive elements in the naked mole-rat genome (25 vs. ~35 % in other murid genomes) could be an artifact of the limitations imposed by the different sequencing and assembly technologies used. Thus, researchers should exercise caution when directly comparing genomes of the naked mole-rat to other species for some of the differences observed may be due to technical artifacts rather than evolutionary changes.

### Estimates of gene content

Approximately, 22,000 genes were identified in the original sequencing (hetGla1) of the naked mole-rat genome (Kim et al. 2011; Bens et al. 2016). This number is similar to that reported in other mammalian genomes. Analysis of gene homology revealed that ~17,000 naked mole-rat genes have a direct ortholog in either human, mouse, or rat. Moreover, when comparing annotation data (Kim et al. 2011), the naked mole-rat genome appears to exhibit 93 % synteny to that of the human genome, with strikingly less synteny to that of other rodents (83 % to mouse and 80 % to rat). These differences most likely reflect structural rearrangements when compared to the murid common ancestor. Syntenic comparisons to mammals identified 750 gained genes and 320 lost genes in the naked mole-rat (Kim et al. 2011). Further analysis of these data found 66 additional genes present in naked mole-rat that are absent in 11 other mammals. Since these genes have no known homologs, their function is currently unknown.

The second sequencing and gene annotation (hetGla2) effort (Keane et al. 2014) identified ~42,000 coding sequences, of which ~13,000 had a best reciprocal BLAST hit to guinea pig, mouse, or human. Several thousand other coding sequences exhibited high-quality one-way alignments.

**Fig. 2:**
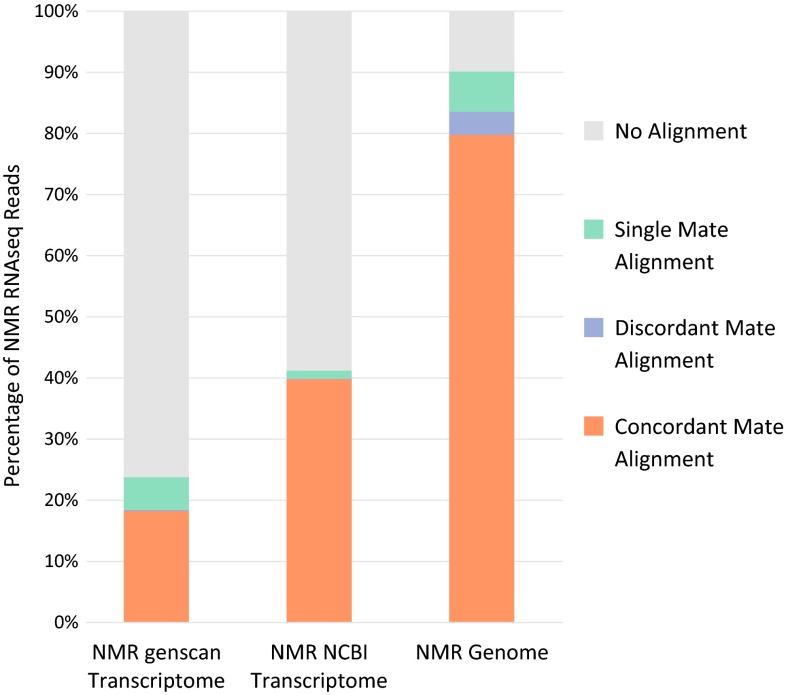
Alignment rates of naked mole-rat RNA-sequence data to its transcriptomes and genome. Paired-end RNAseq data measured from a mixed pool of 7 naked mole-rat tissues (Kim et al. 2011; SRA: SRS213856) were aligned using Bowtie2 (Ben Langmead and Salzberg 2012) to the naked mole-rat transcriptome derived from genscan annotations (Burge and Karlin 1998), naked mole-rat transcriptome derived from NCBI annotations (Keane et al. 2014), and the entire naked mole-rat genome (hetGla_female_1; Keane et al. 2014). Recent NCBI annotations produce the highest fraction of transcriptome alignments; however, ~40–50 % of RNAseq reads align to the genome, but not the annotated transcriptome. Similar distributions of alignment rates were observed with other alignment methods (not shown)

Notably, current annotations of the naked mole-rat genome were generated bioinformatically using sequence homology to well-curated genomes (e.g., mouse and human). Yet, approximately 50 % of the RNAseq reads from the naked mole-rat align to the unannotated parts of its genome [Fig. 2; data from Kim et al. (2011)], suggesting that a significant fraction of the transcriptome is escaping gene annotation. This limits transcriptome comparisons only to the recovered orthologs, preventing the identification of novel naked mole-rat genes.

As a potential step forward, a transcriptome assembly based framework (FRAMA) was created that annotated genes from high coverage RNAseq data collected in multiple tissues (Bens et al. 2016), and this seems to be a promising direction in making subsequent analyses more reliable. Using human gene annotations from the highly curated human genome as a point of reference, FRAMA identified ~17,000 corresponding naked mole-rat genes, indicating that roughly 88 % of human genes have a naked mole-rat ortholog. This suggests that despite a ~90 mya of divergence, such an approach could perform well for ortholog inference (Bens et al. 2016).

**Fig. 3:**
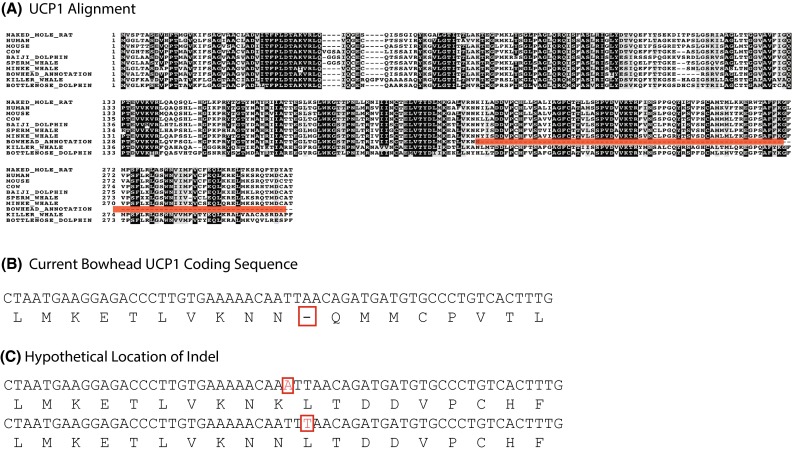
UCP1 alignment in selected cetaceans, and nonaquatic dwelling mammals. **a** Alignment of the UCP1 protein. **b** Bowhead whale UCP1 mRNA revealing a stop codon. **c** Potential location of the InDel reveals that a single-nucleotide change could correct the sequence to match that of other mammals

Nevertheless, currently, the best genome annotation algorithms can create incorrect annotations of a genomic sequence even in well-studied genomes, such as humans and mice; this is obviously exacerbated in less well-studied, more exotic species. For instance, miscalled exons in the annotation can cause frameshifts, leading to erroneously truncated genes (Zhang et al. 2012). Such may be the case with UCP1 in the cetaceans. It was previously reported that whales (Minke, fin, bowhead, and sperm) all had a premature stop codon in the C-terminal region when compared to terrestrial mammals (Keane et al. 2015). As with the naked mole-rat, changes in UCP1 in these cetaceans potentially contribute to altered mass-specific metabolic rates and thermogenic function, and this change can be inferred to be a “longevity assurance mechanism” that contributes to increased lifespan of these organisms (Keane et al. 2015). However, the most recent data available on NCBI for multiple cetaceans, including dolphins and whales, as well as naked mole-rats, cows, humans, and mice, show that all of these, with the exception of the bowhead whale, have full lengths coding sequences for the UCP1 protein (Fig. 3a). However, this truncated amino acid sequence may result from the loss of one nucleotide in the bowhead whale UCP1 sequence (Fig. 3b). The stop-codon in the genomic sequence may however be a sequencing error. For example, if a “T” was missed in the Illumina data but hypothetically reinserted bioinformatically, the correct translation for a full length UCP1 gene can occur (Fig. 3c). The type of DNA polymerase employed in the ‘sequencing by synthesis’ technique (e.g., Illumina) is known to introduce this type of error in homopolymeric regions (Schirmer et al. 2016). More work is needed to determine if this is indeed an error or a real species difference. As shown in Fig. 3, this gene is well conserved among all the mammals examined beyond the region of the putative stop codon, suggesting that this is not a pseudogene. The presence of the stop codon must be confirmed by additional orthologous genomic methods with different error profiles. This is a cautionary tale and illustrates the necessity of both improved manual curation and whole-genome sequences.

### Rapidly and slowly evolving genes

Interspecies differences in mutation rates are generally thought to indicate different evolutionary pressures, while loss of function and frame-shift mutations are indicative of the loss of selection pressure on the genome to maintain functional protein forms. Sequences with greater accumulation of nonsynonymous differences (*K*_a_) relative to synonymous differences (*K*_s_) may highlight key pathways that are under positive selection pressure (Wagner 2002). Thus, *K*_a_/*K*_s_ is a convenient marker of selective pressure. Homologous genes with a *K*_a_/*K*_s_ ratio greater than 1 are considered to be under positive selection pressure.

**Fig. 4:**
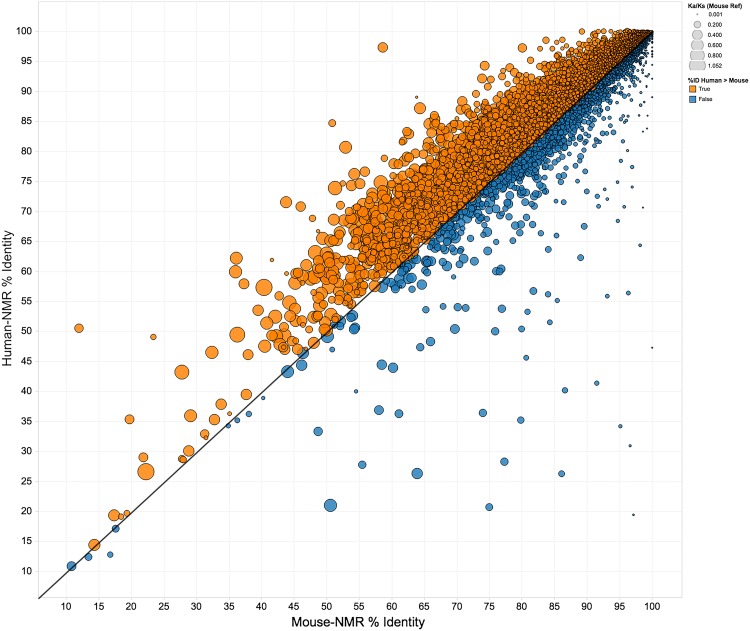
Divergence between naked mole-rat, mouse, and human protein coding sequence. Outliers from the diagonal represent more than expected accumulation of amino acid changes. Naked mole-rat proteins that show greater similarity to those of humans than to those of mice (as indicated by an increase in Ka/Ks ratio relative to mouse) are colored in *orange* and lie above the line of identity

Previous studies analyzing orthologous genes of terrestrial and subterranean species revealed that the nucleotide substitution rate of coding sequences was markedly lower in those species that lived below ground (Du et al. 2015; Shao et al. 2015). In general, the rates of both synonymous and nonsynonymous differences have slowed down in the naked mole-rat relative to both the mouse and guinea pig (Du et al. 2015). This apparent decrease in evolutionary rates of change may be associated with life in a stable and protected environment and may be due to a longer generation time. In addition, the naked mole-rat’s Ka/Ks ratio is higher than that of the mouse and can be attributed to the lack of purifying selection due to a smaller population size (Kim et al. 2011). Intriguingly, the naked mole-rat genes showed greater similarity with humans than with mice (Fig. 4). Note, however, despite a high level of synteny overall, only ~60 % of the annotated naked mole-rat genes have a good one-to-one ortholog to both human and mouse (Keane et al. 2014), suggesting that this comparison is somewhat biased (Fig. 4).

We performed full Smith-Waterman alignment (Smith and Waterman 1981; Zhao et al. 2013) of the naked mole-rat transcriptome (derived from genscan annotations) to those of the mouse [mm10 UCSC annotations] and the human [hg19 UCSC annotations]. This allows for a more forgiving alignment and is generally considered the gold standard. Despite the limitations of these analyses, the naked mole-rat transcriptome showed double the alignment rates and greater parallels with the human transcriptome than that of mouse.

The first study on the naked mole-rat transcriptome was completed prior to the publication of the genome and described only the genes that were upregulated in the naked mole-rat when compared to the C57BL/6 mouse for genes that are expressed at a lower level may simply reflect poor homology with the mouse genome (Yu et al. 2011). Surprising similarities are evident among the transcriptomes of subterranean rodents *Spalax galili*, *Heterocephalus glaber*, and *Fukomys damarensis* (Bennett and Faulkes 2000; Begall et al. 2007; Davies et al. 2015). Several hundred genes are under positive selection in all three of these phylogenetically distinct subterranean rodent species, many of which are likely to reflect shared adaptations to a subterranean lifestyle (i.e., life under hypoxic conditions, resistance to cytotoxins, and cancer).

On the other hand, multiple pathways associated with proteostasis, genomic stability, and cell cycling are more similar between the naked mole-rat, other nonmuroid rodents (including above-ground dwelling rodents, i.e., guinea pigs, chinchillas, squirrels, and jerboas) and humans than among laboratory mice and rats and these species. These pathways likely underwent accelerated evolutionary change in both rats and mice and much slower divergence in the nonmuroid rodents and humans (Vinogradov 2015). This divergence of the Muridae (rats and mice) from the ancestral lineages is thought to reflect a relaxation in purifying selection of these pathways essential for both genomic and proteomic stability (Vinogradov 2015). This may explain the greater propensity for cancer and a shorter than expected lifespan predicted on the basis of their body size for muroid rodents (Hulbert et al. 2007).

Despite the caveats outlined above, comparing the long-lived naked mole-rat to its similarly sized, shorter-lived mouse counterpart reveals a number of phenotypic differences between species (Table 4) that appear to be likely candidates pertinent to both aging studies and key age-associated diseases. These interspecific differences are linked to oxidative stress response, genomic maintenance, and proteostasis.

**Table 4:** Phenotypic differences between naked mole-rats and mice

Phenotype/Pathway	Naked mole-rat vs. Mouse (C57Bl/6)
Oxidative damage	**↑**
Antioxidant levels	**→**
Cytoprotective [NRF2] signaling activity	**↑**
Genome maintenance (DNA repair)	**↑**
Cancer incidence	**↓**
Telomere length	**↓**
Telomerase	**↓**
Tolerance of hypoxia and hypercapnia	**↑**
mTOR signaling	**↓**
Proteome maintenance	**↑**
Autophagy	**↑**
Proteasome activity	**↑**

Naked mole-rats are extraordinarily long-lived compared to the similarly sized mouse. Many previous studies have started to characterize aging-related phenotypes in the naked mole-rat. Compared to shorter-lived mice (i.e., C57Bl/6), naked mole-rats are cancer resistant and tolerant of exogenous stressors including hypoxia and hypercapnia. They also have elevated proteome and genome maintenance, autophagy, and proteasome activity levels compared to mice. Strikingly, they have high levels of oxidative damage even from a young age compared to mice, and both species have similar levels of antioxidant enzymes (i.e., SOD). Despite this, naked mole-rats have high constitutive levels of cytoprotective NRF2-signaling activity. This may be one critical pathway that contributes to their lengthened healthspan and lifespan

## Genomic and transcriptomic comparisons of specific biochemical pathways

### Oxidative damage

Transcriptome analyses revealed that mitochondrial, oxidoreduction, and fatty acid metabolism pathways are upregulated in naked mole-rats with respect to mice (Yu et al. 2011). Paradoxically, tissues from captive naked mole-rats have high levels of oxidative damage to lipids, proteins, and DNA from an early age (Andziak and Buffenstein 2006; Andziak et al. 2006; Buffenstein et al. 2008). Not surprisingly, these damage levels are paired with a lackluster antioxidant defense. Naked mole-rats have similar levels of key antioxidant enzymes to those of mice, with the exception of cytosolic glutathione peroxidase [cGPX], which is 70-fold lower in naked mole-rats (Andziak et al. 2005; Lewis et al. 2013). Transcriptomic studies confirm that both *Gpx1* and *Gpx4* are lower in naked mole-rats compared to mice (Yu et al. 2011). Additionally, two of the six peroxiredoxins (*Prdx2* and *Prdx5*) are expressed at lower levels than observed in mice both at the transcript and protein level (Fang et al. 2014). Despite the low levels of antioxidant expression, naked mole-rats (and other long-lived rodents including the blind mole-rat) do have elevated levels of other cytoprotective pathways (e.g., NRF2, molecular chaperones) compared to the mouse (Edrey et al. 2014; Rodriguez et al. 2014; Lewis et al. 2015).

### Genome maintenance and cancer resistance

A global comparison of previously known human cancer-associated oncogenes and tumor suppressors in the naked mole-rat genome did not reveal any striking difference in copy number variation (MacRae et al. 2015). Rather, a lower frequency of mutations has been observed in a subset of 518 genes linked to genome maintenance in naked mole-rats, mice, and humans (MacRae et al. 2015), which may suggest that positive selection is acting on this gene group. Consistently, many of the enzymes directly pertinent to regulating DNA repair are expressed at higher levels in liver, brain, and testes than observed in mice (Kim et al. 2011; MacRae et al. 2015), including enzymes involved in tumor suppression (e.g., TP53), base excision repair, mismatch repair, and nonhomologous end-joining (MacRae et al. 2015). It has been thought that increased levels of p16 were associated with the “contact inhibition phenomenon” observed in naked mole-rat primary cultures of dermal fibroblasts in some laboratories (Seluanov et al. 2009). Further analysis of the INK4 locus revealed unique splicing patterns that encodes an additional protein, pALT^INK4a/b^, which may contribute to cell cycle regulation and cancer resistance in naked mole-rats (Tian et al. 2015). In-depth genome analysis revealed that the p16INK4a may be altered in structure, producing a smaller protein with earlier stop codons (14-kDa) (Kim et al. 2011), that may also impact cell cycle progression and tumorigenesis.

Intriguingly, the distantly related, but nonetheless cancer-resistant blind mole-rat (*Spalax ehrenbergi*; Table 1) has purportedly evolved different anticancer mechanisms to that of the naked mole-rat (Manov et al. 2013). p53 in *Spalax* differs from that of most mammals with a change in amino acid sequence akin to a specific mutation frequently found in tumors (Shams et al. 2013). This subtle difference in p53 protein is not without major consequence; it alters the ability of this species to induce apoptosis and enhances the immune-inflammatory processes promoting interferon B1-induced necrosis (Shams et al. 2013). In contrast, the naked mole-rat p53 does not show this potentially beneficial mutation (Gorbunova et al. 2014), but is more similar to that of humans than that of mice and rats with similar proline-rich domains that reportedly have been subject to positive selection (Keane et al. 2014).

### Telomeres and telomerase

A number of genes in the naked mole-rat genome have undergone positive selection, including genes involved in the function and regulation of telomerase (*Tep1* and *Terf1*) (Yu et al. 2011). Telomere shortening is thought to play a pivotal role in aging and as they reach critically short length, cells enter a senescent state. Studies evaluating telomere length and telomerase activity have produced equivocal and contradictory findings [(Seluanov et al. 2007; Gomes et al. 2011); Yang, Hornsby and Buffenstein *pers. com*]. Gomes et al. 2011 reported the telomeres of naked mole-rats to be much smaller (one-third to one half) than those of lab mice and rats, and of similar length to those in humans (Gomes et al. 2011), with telomerase activity in cultured naked mole-rat dermal fibroblasts just one-third of what is observed in mouse fibroblasts (Gomes et al. 2011). The naked mole-rat telomerase genes have unique polymorphisms and promoter structure (compared to guinea pigs and humans) (Evfratov et al. 2014). *Tep1*, *Terf1*, and other genes that regulate telomerase activity likely also contribute to the slow-aging and cancer-resistant phenotype of the naked mole-rat (Yu et al. 2011).

### Tolerance of hypoxia, hypercapnia, ammonia, and pain

Naked mole-rats (as well as other subterranean mole-rat species) evolved to live successfully in a sealed maze of hypoxic underground tunnels where gas exchange through soil is poor. Transcriptomic differences relative to mice and rats are evident in all three subterranean species of mole-rats even under normoxia (Fang et al. 2014). For example, hemoglobin α (*Hba1* and *Hba2*) and neuroglobin (*Ngb*) are more highly expressed in the mole-rats (Avivi et al. 2010; Fang et al. 2014). Both naked mole-rat and guinea pig hemoglobin α share a unique amino acid change (Pro44His) thought to convey better tolerance of hypoxia in the low-oxygen atmospheres encountered underground and at high altitudes, respectively (Fang et al. 2014). Hypercapnia is deadly to most mammals and generally evokes considerable pain; increased levels of CO_2_ turns air acidic, stimulating pain receptors and the burning sensation in nasal passages and eyes (Brand et al. 2010). The lack of pain response in naked mole-rats is attributed to motif changes in the Na(V)1.7 sodium channel (*Scn9a*), a feature shared with Damaraland and blind mole-rats, as well as other species that are chronically subjected to similar atmospheric conditions (Fang et al. 2014), as well as the lack of expression in substance P and calcitonin gene-related gene peptide (*Cgrp*) (Park et al. 2008; Park and Buffenstein 2012). This difference in the sequence, and most likely subsequent negative regulation, could account for their indifference to chemical induced pain (Kim et al. 2011; Park and Buffenstein 2012).

### Insulin and mTOR signaling

The insulin and mTOR pathways are considered key players in mouse and human aging, with downregulation in these pathways and/or altered signaling through mutations of receptors linked to increased longevity (Selman et al. 2008; Selman and Withers 2011; Lamming and Sabatini 2011; Johnson et al. 2013; Lamming et al. 2013; Mulvey et al. 2014). Transcriptomic analyses revealed that the naked mole-rat showed a divergent sequence of the insulin β-chain, similar to that of the guinea pig and other close relatives, i.e., hystricognath rodents (Opazo et al. 2005). Moreover, RNAseq using both liver and brain tissue taken from nonfasted, non stressed animals show that many components of the insulin and mTOR pathways are downregulated and might be indicative of slower growth rates (Kim et al. 2011). Both insulin and insulin receptor gene expression are attenuated, in addition to insulin receptor substrate 1 (*Irs1*). Furthermore, *Pi3* *k* isoforms are also downregulated (with the exception of *Pik3cb*) (Kim et al. 2011), as well as *Igf1* and *Igf1* receptor (*Igf1r*) (Kim et al. 2011). Alternatively, insulin like growth factor 2 (*Igf2*) transcript levels are high (Kim et al. 2011). *Igf2* has high homology with insulin and is commonly expressed at high levels in utero with levels dropping dramatically after birth (Lui and Baron 2013). *Igf2,* and its binding protein *Igf2bp2*, are retained at high levels in adult naked mole-rats (Kim et al. 2011), thereby maintaining a neonatal like mode of glucose handling and most likely remaining highly sensitive to different nutrient signals. The *Igfbp* protease, pregnancy-associated plasma protein-A (*Papp*-*a*), known to modulate mouse lifespan, reportedly has a different sequence in the naked mole-rat to that of mice and is also constitutively expressed at low levels, giving rise to abnormal glucose tolerance tests (Kramer and Buffenstein 2004; Brohus et al. 2015) and raising the possibility that *Papp*-*a* may play a role in naked mole-rat longevity.

### Proteome maintenance

The observed decline in nutrient-sensing gene expression described above may have a profound effect on protein translation and turnover. The proteostasis system of the naked mole-rat is significantly more robust than that of mice (Pride et al. 2015). Naked mole-rats exhibit greater translational fidelity apparently without a reduction in translation rate (Azpurua et al. 2013); their proteins also appear to be more resistant to oxidation, heat, and urea (Perez et al. 2009). This greater structural stability is maintained with age (Perez et al. 2009). Autophagy is also elevated and sustained during aging in the naked mole-rat (Rodriguez et al. 2011; Pride et al. 2015) and double-membrane autophagosomes have been observed in higher numbers in multiple tissues of naked mole-rats (Zhao et al. 2014) compared to mice. Proteasome activity in multiple tissues was found to be significantly higher in naked mole-rats than in shorter-lived mice (Perez et al. 2009; Rodriguez et al. 2012; Edrey et al. 2014) and also exceptionally resistant to inhibition by competitive inhibitors MG-132 and bortezomib and oxidative stress (Rodriguez et al. 2014). Interestingly, it appears that the African and Middle Eastern mole-rats have higher levels of proteasome activity than above-ground-dwelling rodents, and that the level of chymotrypsin and caspase-like activity in muscle tissues significantly correlate well with maximum lifespan (Rodriguez et al. 2016).

## Challenges of comparative genomics

Multiple mammalian genomes have been sequenced and assembled in the recent decades. However, the information that would enable robust comparative genomic analyses is still sparse. As of today, only 19 rodent genomes have been assembled with varying levels of quality, and most sequenced species are only distantly related to the naked mole-rat. The ideal comparative genomic study would include a large number of species as well as multiple individuals within each species to account for intraspecific genetic variation. This limitation in numbers of genomes and their disparate quality makes linking variation in sequence to a specific phenotype more of an anecdotal, rather than data-driven, task.

Comparative genomic studies assessing interspecific differences in genomic data sets present numerous problems. Of critical importance is the choice of the reference genome, with the trade-off being whether to choose a closely related species that is not well annotated or that of a more distantly related species that has been extensively studied and is well annotated. For example, while mice and humans have been extremely well studied, they diverged from mole-rats ~70 and ~90 mya respectively (Kim et al. 2011). The genomes of the more closely related guinea pig (divergence of ~39.5 mya) and the Damaraland mole-rat (divergence of ~31.2 mya) have also been assembled, albeit with lower quality and poorer annotation than that of mice and humans. Possibly because of extensive deep sequence analyses of the human genome, the naked mole-rat genome shows the best homology to that of humans, rather than with other rodents.

In species with poor coverage or poorly annotated genomes, it is particularly difficult to identify genes that are truly under-expressed, orthologs, and/or splice variants. Data analyses are thus limited to those genes with unambiguous annotation. Focusing comparisons on more closely related species could control for much of the confounding genetic diversity. For example, the recent paper comparing transcriptomes of nine African mole-rat species (Davies et al. 2015) is probably the best comparative genomic study using the naked mole-rat to date. This genome-wide screen suggested that genes related to tumor suppression, telomere regulation, cell division, DNA repair, and stress response were under positive selection in the African mole-rat clade (Davies et al. 2015). This provides further mechanistic insight into what may contribute to their notable cancer and stress resistance, and whether these gene expression patterns are unique to the naked mole-rat or shared among closely related species.

Given the large evolutionary distance of the genomes usually compared, finding the causal variation for a phenotype has to be hypothesis driven and requires careful scrutiny and interpretation, for observed species differences likely reflect more about their evolutionary history, ecophysiological traits, or divergent phylogeny. This would certainly be the case if one compared the genomes of the long-lived Bowhead whale directly to the long-lived naked mole-rat. While it is tempting to force hypotheses to fit a priori predictions, this approach often may give rise to spurious “just-so stories.” Two clear examples were recently highlighted: In the original naked mole-rat genome paper (Kim et al. 2011) the “hairless” phenotype of naked mole-rats was attributed to a substitution of conserved amino acid in the identified hair growth associated protein (HR). This interpretation was based on findings that similar mutations in this particular codon cause hair loss in mice, rats, and humans. However, two other hystricognath rodents, namely the Damaraland mole-rat and guinea pig, share this mutation in the HR gene, yet have hairy coats (Delsuc and Tilak 2015). It therefore becomes clear that differences in the mole-rat and mouse/human HR gene simply reflect a phylogenetic divergence from mice and men (Delsuc and Tilak 2015; Davies et al. 2015).

The conclusion concerning hyaluronan synthase 2 (*Has2*) is another such example. Differences in *Has2* have been used as a causal explanation for the extraordinarily low incidence of cancer in naked mole-rats (Seluanov et al. 2009; Tian et al. 2013). Unique amino acid residues in this enzyme reportedly result in the synthesis of a higher molecular weight hyaluronan in naked mole-rats than in mice (Tian et al. 2013). While the *Has2* sequence is unique to the naked mole-rat, extending comparisons to a wide range of other species showed that some of the proposed mutations are shared with several species, including guinea pigs. These mutations do not always result in cancer resistance (Faulkes et al. 2015), although their specific functional ramifications are unknown. Interestingly, high molecular mass hyaluronan is also reportedly expressed in the blind mole-rat (*Spalax galili*) (Tian et al. 2013) despite the fact it does not share any of the significant mutations observed in the naked mole-rat yet reportedly is resistant to cancer (Manov et al. 2013).

### Correlation of phenotype and genotype

As discussed above, one of the confounding factors for the comparative genomics analyses is poor definition of the phenotype to be explained. Longevity itself is not a well-defined phenotype, but a byproduct of a myriad of beneficial phenotypes and/or the absence of detrimental phenotypes including cancer resistance and better cellular and proteome maintenance. Genomics, overall, is still a descriptive science when comparing one entire genome to another the data will be overwhelming and less likely to identify or target specific mechanisms. We suggest that defining the phenotypes conserved between long-lived species (i.e., diverged from short-lived species) could be a major step forward. For instance, when the phenotype is well defined, as in the case of different rRNA processings in naked mole-rats that results in a different structure of the 28S ribosomal subunit (Azpurua et al. 2013; Fang et al. 2014), the comparisons are limited to a subset of genes and suggest meaningful genotype-phenotype connections.

**Fig. 5:**
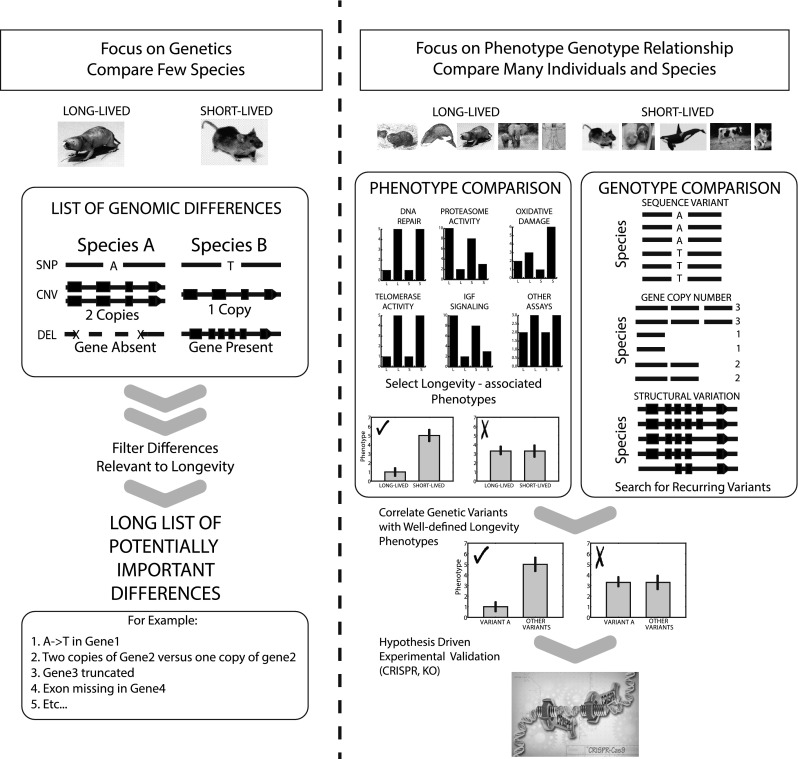
A summary of future project directions. Current comparative genomic studies usually compare 1 long-lived and 1 short-lived species (i.e., the naked mole-rat vs. the mouse) and result in a large number of genes that may or may not involved in healthspan and/or longevity that have not been experimentally validated. We propose that comparing the genomes of a large number of both long- and short-lived species and focusing on specific phenotypes that may contribute to the extended healthspan and lifespan would yield more focused and meaningful genome data. These results would then be confirmed through hypothesis-driven experimental validation to determine further impact on longevity

In another example, data across many species (including worms and flies) indicate that proteasome function is critical to lifespan, and may also promote extended healthspan and longevity-related phenotypes (Ghazi et al. 2007; Tonoki et al. 2009; Kruegel et al. 2011). Previous data have also shown that increased proteasome activity is observed in naturally long-lived species (Pickering et al. 2015), including the naked mole-rat (Rodriguez et al. 2012, 2016), although we know nothing about the genetic mechanisms behind these phenotypes. By interrogating the genomic data for specific, proteasome-related genes (Table 5), we can get a more in-depth picture of not only those specific gene sequences, but also how small differences may impact this longevity-related phenotype, and experimentally test causality of these genetic differences, using tools like CRISPR, in vitro and/or in vivo (Fig. 5).

**Table 5:** Hypothesis-driven approaches to comparative genomic study design

Organism	Condition	Phenotype	References
*S. cerevisiae*	ubr2∆	Increased replicative lifespan	Kruegel et al. (2011)
*S. cerevisiae*	rpn4∆	Decreased replicative lifespan	Kruegel et al. (2011)
*D. melanogaster*	RPN11 overexpression	Increased lifespan	Tonoki et al. (2009)
*C. elegans*	Knockdown of rpn-1, rpn-3, rpn-6, rpn-7, rpn-8, rpn-9, rpn-11, rpt-1, rpt-4, rpt-5, rpt-6, pas-5, pas-6, pbs-2, pbs-3, pbs-4, pbs-5, or pbs-7	Decreased lifespan	Ghazi et al. (2007)
*C. elegans*	pbs-5 overexpression	Increased lifespan	Chondrogianni et al. (2015)
*M. musculus*	Pac1 knockout	Early embryonic lethality, decrease in free 20S proteasome, premature senescence	Sasaki et al. (2010)
*M. musculus*	Psmc1 knockdown in brain	Protein aggregation and neurodegeneration	Bedford et al. (2008)
*H. sapiens* (WI-38)	Lactacystin treatment	Reduces RLS	Torres et al. (2006)
*H. glaber*	Unknown	Unknown	Unknown

Perturbations to genes related to the proteasome, as well as the proteasome itself, result in changes to lifespan and healthspan of a variety of organisms, including yeasts, worms, flies, mice, and even humans. The long-lived naked mole-rat also has elevated proteasome activity, but we have little information about the genes involved. Thus, by interrogating the genome specifically for proteasome-related genes, we can study these genes more in depth and compare with other species, to identify beneficial (or detrimental) mutations or polymorphisms. Differences in gene/protein sequence can then be studied in vitro or in vivo to identify causal variants. [References in Table: Torres et al. (2006), Bedford et al. (2008), Sasaki et al. (2010), Chondrogianni et al. (2015)]

Another approach to better define the phenotype of increased lifespan includes measuring the transcriptomic and proteomic responses of long-lived and short-lived species to a similar battery of perturbations. For instance, to better define the phenotype of cancer resistance of the naked mole-rat, one could examine differences in the transcriptional response to cancer-causing agents between the cancer-resistant and cancer-prone species. This approach will enable identification of genes, pathways, or regulatory molecules that are differentially regulated between long-lived and short-lived species, focusing the comparative genomic query to find the variation that is causal for this change.

Finally, genetic differences that can generate significant phenotypic variation between even closely related species are postulated to be a result of regulatory, and not coding, sequence changes [reviewed in Varki and Altheide (2005)]. Better annotation of regulatory sequences in the species of interest (e.g., chromatin profiling) could make linking the genetic variation to phenotypic difference easier.

In summary, technological improvements will provide us with genomic data of far greater resolution than what is available today. Similarly, more carefully controlled comparative studies using state-of-the-art bioinformatics tools will yield high-quality, unambiguous data. As a direct result of these improvements, greater insights into the unique traits of naked mole-rat will become possible, particularly when combined with phenotypic profiling. Improving access to the secrets within the naked mole-rat genome will elucidate the mechanisms naked mole-rats employ to resist the vagaries of aging and prevent age-associated diseases from gaining hold.
